# Interviewer‐assisted administration of patient‐reported outcome measures with intentional interviewer influence consistently produces more favourable scores than self‐administration after total knee arthroplasty: A prospective within‐subject comparative study

**DOI:** 10.1002/jeo2.70899

**Published:** 2026-10-09

**Authors:** Mara Hold, Sophie Berg, Alexander Derksen, Jan Dirk Kiene, Mats Zander, Marco Haertlé, Spiros Tsamassiotis, Henning Windhagen, Lars‐Rene Tuecking

**Affiliations:** ^1^ Department of Orthopaedic Surgery, DIAKOVERE Annastift Hannover Medical School Hannover Germany

**Keywords:** interviewer bias, patient‐reported outcome measures, questionnaire administration, total knee arthroplasty, value‐based medicine

## Abstract

**Purpose:**

Patient‐reported outcome measures (PROMs) are key instruments for evaluating outcomes after total knee arthroplasty (TKA), but differences across studies suggest that methodological factors, including interviewer bias, may affect their validity. The purpose of this prospective within‐subject study was to determine whether intentionally influenced interviewer‐assisted administration produces more favourable Western Ontario and McMaster Universities Osteoarthritis Index (WOMAC), Forgotten Joint Score (FJS) and Oxford Knee Score (OKS) scores than self‐administration after TKA.

**Methods:**

Three months after TKA, consecutive eligible patients completed the WOMAC, FJS and OKS twice: first independently at home and later in an intentionally influenced interviewer‐assisted format during routine follow‐up. During interviewer‐assisted administration, questions were clarified when needed, and when patients were unsure between two response options, the more favourable answer was suggested. Paired *t* tests and standardized paired effect sizes (*dz*) were calculated.

**Results:**

Sixty patients with complete paired assessments were included (41 women and 19 men; mean age 63.7 ± 8.2 years). Interviewer‐assisted administration yielded mean differences of 35.9 ± 17.7 points for the FJS (*p* < 0.001; *dz* = 2.03), 6.8 ± 4.7 points for the OKS (*p* < 0.001; *dz* = 1.44) and 8.4 ± 10.9 points for the WOMAC (*p* < 0.001; *dz* = 0.77), consistently indicating more favourable scores.

**Conclusions:**

PROMs after TKA are strongly influenced by the mode of administration. Interviewer‐assisted administration involving intentional influence resulted in systematically more favourable scores across all questionnaires. For meaningful comparison of PROM‐based outcomes, the mode of administration and any interviewer guidance should be standardized or at least clearly reported.

**Level of Evidence:**

Level III.

**Abbreviations**
FJS: Forgotten Joint Score
OKS: Oxford Knee Score
PROMs: patient‐reported outcome measures
TKA: total knee arthroplasty
WOMAC: Western Ontario and McMaster Universities Osteoarthritis Index

## INTRODUCTION

Patient‐reported outcome measures (PROMs) are well‐established tools for evaluation of patient‐centred outcomes in clinical research and routine care. By capturing the subjective assessment of symptoms, function and quality of life, PROMs provide essential information that complements clinical, radiographic or surgical parameters [6, 32]. Their use in clinical research has increased during the past 15 years [21], and PROMs are increasingly integrated into national arthroplasty registries for large‐scale outcome assessment and international benchmarking, although comparability remains methodologically challenging [29]. Significant differences in mean PROM results for total knee arthroplasty (TKA) in national and international comparisons, despite comparable alignment strategies and patient populations, suggest that there are more factors that influence outcome parameters [18], including the mode of administration. Typically, PROMs are collected using standardized questionnaires, administered either independently by patients, on paper, digitally or via mail, or through telephone interviews or guided completion with trained staff [1, 19, 20, 22]. However, evidence from social sciences indicates that the mode of administration can lead to systematic response variations and thereby influence outcome validity [31, 37]. This is particularly relevant when PROMs are used as primary endpoints. Beyond clinical research, PROMs are increasingly discussed as potential instruments for value‐based healthcare and quality‐based reimbursement models [10, 34]. Examples include the Centers for Medicare and Medicaid Services (CMS) Total Hip Arthroplasty/TKA Patient‐Reported Outcome–Performance Measure programme in the United States [27] and emerging PROM‐linked reimbursement contracts in Germany [7]. Therefore, methodological factors that may systematically influence PROM results, such as differences in administration format, may become increasingly relevant for both scientific comparability and healthcare evaluation.

For international use, PROMs are frequently translated and culturally adapted using standardized procedures, including forward and backward translation, expert review, pretesting and psychometric validation. Although these processes aim to ensure conceptual equivalence [36], subtle semantic differences may persist, particularly for abstract or cognitively framed constructs. Such nuances may become relevant when additional explanations are provided during interviewer‐assisted administration.

In orthopaedic surgery, several studies have addressed interviewer bias. Hammarstedt et al. reported significantly better PROM scores when questionnaires were completed during a telephone interview compared with e‐mail submission or in‐person consultation after hip arthroscopy [13]. Similarly, Höher et al. demonstrated superior Lysholm scores when administered jointly during an outpatient visit rather than completed independently beforehand [15]. In contrast, Lopes et al. found no significant differences between independent and interviewer‐guided PROM administration in patients treated for rotator cuff pathology [22]. These findings suggest that interviewer interaction may modify patient responses, although its effects appear inconsistent and likely dependent on the assessment instrument. Evidence regarding interviewer influence after TKA remains limited. Bellamy et al. compared telephone and face‐to‐face Western Ontario and McMaster Universities Osteoarthritis Index (WOMAC) administration on consecutive days and found no significant differences [4]. The purpose of this prospective within‐subject study was to determine whether intentionally influenced interviewer‐assisted administration produces more favourable WOMAC, Forgotten Joint Score (FJS) and Oxford Knee Score (OKS) scores than self‐administration after TKA.

## METHODS

### Study design

This prospective comparative study focused on the influence of the method of PROM administration after TKA. The established knee‐specific outcome instruments, the WOMAC, the FJS and the OKS, were evaluated twice in each patient. The first assessment was performed independently by the patient at home or elsewhere, whereas the second assessment was conducted under the direct supervision and guidance of clinical study personnel in a short time interval.

### Ethics statement

Ethical approval for this study was obtained from the local ethics committee (Nr. 11678_BO_ K_2024). It included the partial disclosure of the study objective and intentional guidance during the second assessment. The specific objective was withheld until completion of both assessments to minimize expectancy‐related response bias. After completing both assessments, participants were formally debriefed and could withdraw their consent and data without disadvantage. Participation or withdrawal had no effect on clinical care.

### Patient characteristics and inclusion/exclusion criteria

As inclusion criteria were defined, all patients who had undergone primary TKA in a single centre performed by two senior arthroplasty surgeons. Exclusion criteria were relevant language barriers, patients who had a legal guardian or equivalent support in daily life or suffered from cognitive impairment that could interfere with questionnaire comprehension. Individuals with chronic pain syndromes were also not considered eligible, as this condition may independently bias responses. All patients were recruited postoperatively within their hospital stay. A physician provided verbal and written information regarding study procedures, and patients were given sufficient time to ask questions. Patients were blinded to the study objective as they were told the study was about early outcomes after TKA. Written informed consent was obtained prior to enrolment.

### PROM instruments and scoring

Validated German‐language paper versions of all PROMs were used. The 12‐item FJS assesses joint awareness and ranges from 0 to 100, with higher scores indicating less joint awareness and therefore a more favourable outcome. The 12‐item OKS assesses pain and function and ranges from 0 to 48, with higher scores indicating less pain and better function. The 24‐item Likert‐format WOMAC comprises pain, stiffness and physical‐function domains. Items are scored from 0 to 4 and summed to a score ranging from 0 to 96, with lower scores indicating less pain, stiffness and functional limitation.

### Data collection procedure

PROM collection took place at 10–12 weeks after surgery. Each patient completed the three PROM questionnaires twice within a short interval of approximately 2 weeks. For the first assessment, patients received paper‐based versions of the WOMAC, FJS and OKS via postal mail 1–2 weeks before their scheduled postoperative follow‐up appointment. Participants completed the questionnaires independently at home without any explanation or supervision. The questionnaires were returned via mail, and their receipt was electronically documented. Questionnaires had to be returned before the consultation to minimize recall bias. The second assessment was conducted shortly after during the routine clinical 3‐month‐follow‐up appointment. At the beginning of the consultation, patients were first asked about their overall satisfaction with the surgical result. A 5‐point Likert scale was used for this purpose. Then the same PROMs were administered again, but under the supervision of a trained medical student (S. B.). The interviewer was not involved in the patients' surgical treatment or routine postoperative care and was trained in the study‐specific administration procedure before data collection (by M. H. and L.‐R. T.). A formal verbatim script was not used. However, the same predefined procedure was applied to all patients. Items and response options were read aloud, while patients could also reread the written questions themselves. The interviewer had access to the previously completed self‐administered questionnaires and was therefore not blinded to the prior scores. This was intentional, as the second assessment was designed as an actively guided situation in which responses could be influenced toward more favourable scores. During interviewer‐assisted administration, response options were intentionally provided in altered order to minimize recall bias from the previously completed questionnaires. When patients expressed uncertainty between two answer choices, the clinically more favourable option was suggested. This was defined as the response indicating less joint awareness for the FJS, less pain or functional limitation for the OKS, and less pain, stiffness or functional limitation for the WOMAC. Items were explained when required, and responses were only recorded once the patient verbally confirmed agreement. Thus, the second administration represented an actively guided scoring situation in contrast to the independent assessment at home. After filling out the forms for the second time, patients were told the real objective of the study. The flow chart of the study is shown in Figure 1.

**Figure 1:**
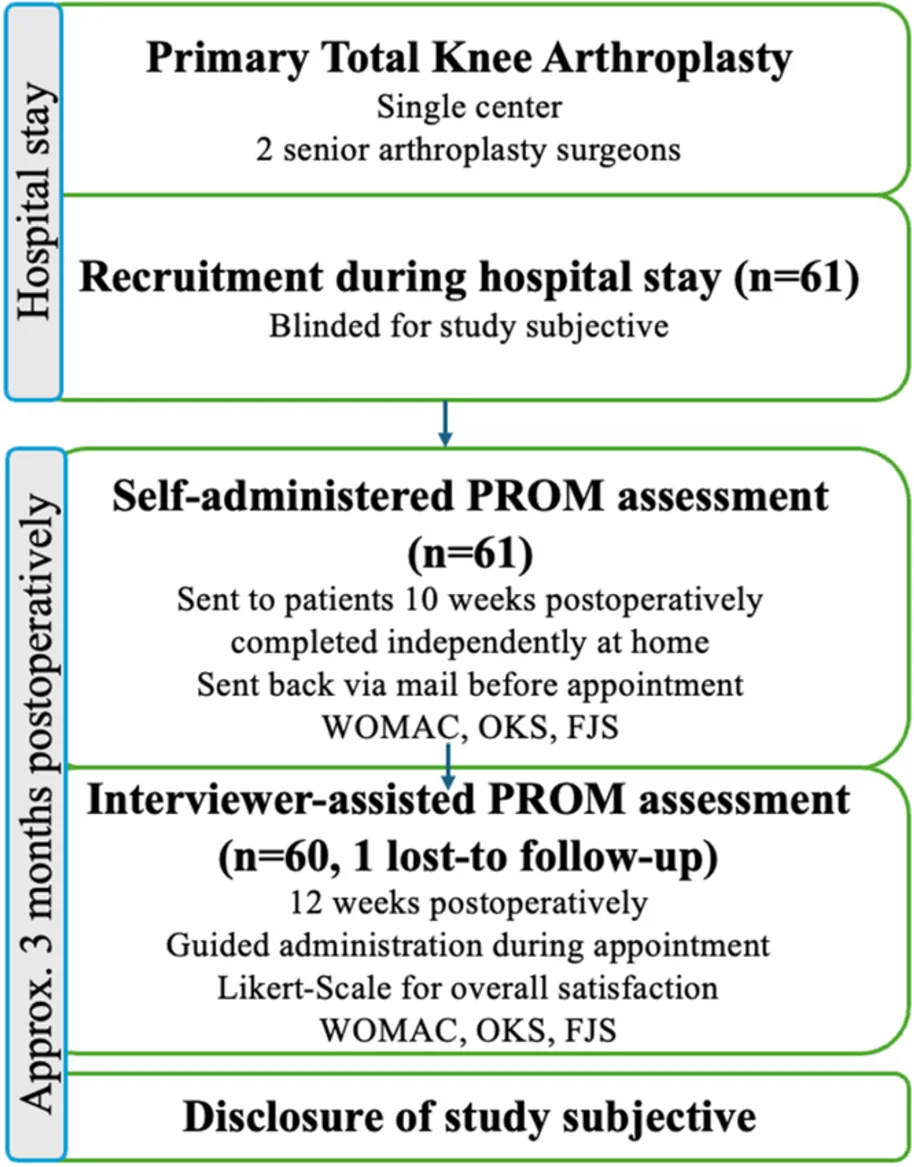
Flow chart illustrating patient recruitment and study timeline. FJS, Forgotten Joint Score; OKS, Oxford Knee Score; PROM, patient‐reported outcome measure; WOMAC, Western Ontario and McMaster Universities Osteoarthritis Index.

### Statistical analysis

Sample‐size planning was based on the expected difference in the total WOMAC score between administration methods based on the study by Bellamy et al. [4]. Assuming a standardized effect size of 0.57, a two‐sided significance level of 0.05 and a power of 0.95, 43 complete paired assessments were required. After accounting for an anticipated dropout rate of 20%, at least 54 patients had to be recruited, and the recruitment target was rounded up to 60 patients.

Only patients with complete data from both assessments were included in the paired analysis. For each questionnaire and each administration method, mean values and standard deviations (SDs) were calculated. Normality of the within‐patient differences was assessed separately for each PROM using the Shapiro–Wilk test. Paired‐sample *t* tests were used for normally distributed differences. For non‐normally distributed differences, a Wilcoxon signed‐rank test was additionally performed as a sensitivity analysis. To investigate a possible systematic effect of the survey method, the differences were stratified according to the postoperative satisfaction level (Likert scale 1–5). For each Likert group and each score, the mean, SD, standard error and 95% confidence intervals (CIs) of the differences were calculated. The standardized paired effect size (*dz*) was determined to quantify the effect strength. Statistical significance was defined as *p* < 0.05. Statistical analyses were performed using R software (R Version 4.3.2; R Core Team, 2023) with the EZR (Easy R) plugin and the ggplot2 package for data visualization.

## RESULTS

### General demographics and clinical characteristics

Patients were recruited postoperatively between January and August 2025. PROM assessments were conducted between March and December 2025 within the predefined postoperative follow‐up window. A total of 61 patients were enroled. One patient was lost to follow‐up before the second assessment, leaving 60 patients available for the paired analysis. No item‐level data were missing among the completed questionnaires; therefore, no replacement or adjustment of missing values was required. Moreover, 19 patients (31.6%) were male, and 41 (68.3%) were female. The mean age at surgery was 63.7 ± 8.2 years, while the mean body mass index was 29.4 ± 4.6 kg/m^2^. The first assessment was completed at a mean of 73.9 ± 7.5 days after surgery (range: 54–106 days), and the second assessment was completed after 91.1 ± 6.5 days (range: 78–120 days). The mean interval between assessments was 17.2 ± 3.0 days (range: 9–25 days).

### Main study findings

All evaluated PROMs differed significantly between self‐administered and intentionally influenced interviewer‐assisted assessment (Table 1). The FJS increased from 25.3 ± 22.4 to 61.2 ± 24.7, yielding a mean difference of 35.9 ± 17.7 points (95% CI 31.4–40.5; *p* < 0.001). WOMAC scores decreased from 22.6 ± 16.4 to 14.3 ± 11.7, corresponding to a mean difference of −8.4 ± 10.9 points (95% CI −11.2 to −5.6; *p* < 0.001). The OKS increased from 29.8 ± 9.2 to 36.6 ± 7.4, with a mean difference of 6.8 ± 4.7 points (95% CI 5.6–8.0; *p* < 0.001). The Shapiro–Wilk test showed no evidence of non‐normality for the FJS (*W* = 0.988, *p* = 0.842) or OKS differences (*W* = 0.974, *p* = 0.239), whereas WOMAC differences deviated from normality (*W* = 0.951, *p* = 0.017). For WOMAC, the Wilcoxon signed‐rank test also demonstrated a significant difference between modes of administration (*p* < 0.001). The standardized effect sizes were 2.03 for the FJS, 0.77 for the WOMAC score and 1.44 for the OKS. For FJS and OKS, the majority of patients exhibited positive differences (interviewer‐assisted minus self‐administered), whereas WOMAC differences were predominantly negative, corresponding to lower symptom scores under interviewer‐assisted assessment (Figure 2). Only a small proportion of patients demonstrated minimal or inverse differences, confirming a consistent administration‐mode effect across the cohort. Within‐subject trajectory plots further illustrated this effect (Figure 3). Stratification by postoperative satisfaction showed that interviewer‐related differences were higher in patients with moderate to high satisfaction (Likert 3–5), as shown in Supporting Information S1: Figure 1. For FJS and OKS, statistically significant differences were observed from Likert 3 upwards, with an increasing effect at higher satisfaction levels. WOMAC differences were largest at moderate satisfaction and remained significant in highly satisfied patients, while no significant differences were detected in patients with low satisfaction (Supporting Information S1: Table 1).

**Table 1:** Mean measurements, SDs and standardized effect size of PROMs.

Questionnaire	Self‐administered	Interviewer‐assisted	Mean difference ± SD	*p* value	95% CI	*dz*
FJS	25.3 ± 22.4	61.23 ± 24.69	35.92 ± 17.68	<0.001	31.4 to 40.5	2.03
WOMAC	22.6 ± 16.41	14.3 ± 11.66	−8.37 ± 10.87	<0.001	−11.2 to −5.6	0.77
OKS	29.78 ± 9.18	36.57 ± 7.4	6.78 ± 4.71	<0.001	5.6 to 8.0	1.44

*Note*: Mean difference was calculated as the interviewer‐assisted score minus the self‐administered score. Positive FJS and OKS values and negative WOMAC values indicate more favourable scores during interviewer‐assisted administration. Data are presented as mean ± SD. *p* values were calculated using paired‐sample *t* tests. For WOMAC, an additional Wilcoxon signed‐rank sensitivity analysis also yielded *p* < 0.001.

Abbreviations: CI, confidence interval; *dz*, standardized effect size for paired samples; FJS, Forgotten Joint Score; OKS, Oxford Knee Score; *p*, probability value; PROMs, patient‐reported outcome measures; SD, standard deviation; WOMAC, Western Ontario and McMaster Universities Osteoarthritis Index.

**Figure 2:**
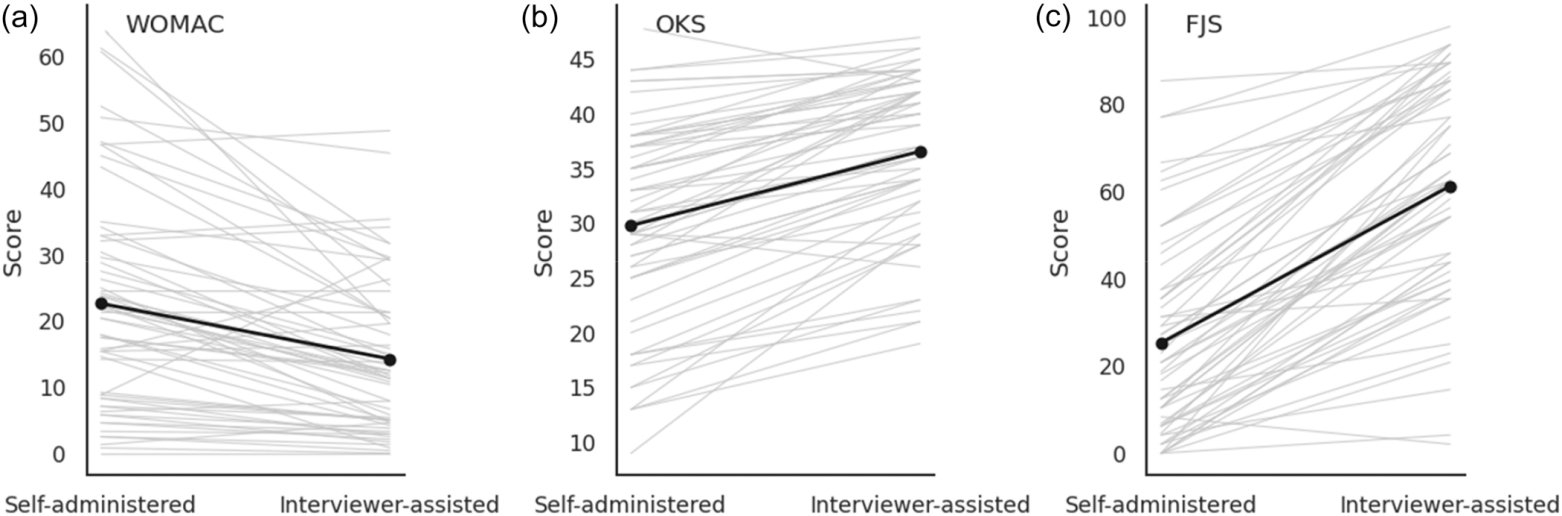
Trajectories of tested PROMs from self‐administered to interviewer‐assisted data collection for WOMAC (a), OKS (b) and FJS (c). FJS, Forgotten Joint Score; OKS, Oxford Knee Score; PROMs, patient‐reported outcome measures; WOMAC, Western Ontario and McMaster Universities Osteoarthritis Index.

**Figure 3:**
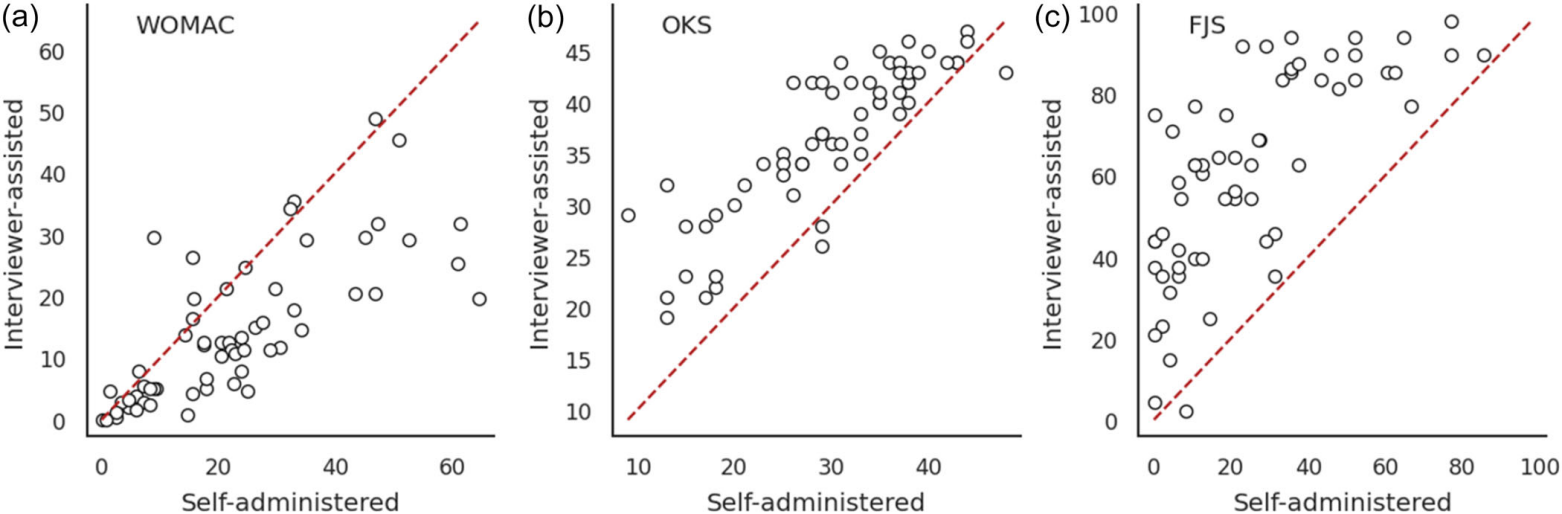
Scatterplots comparing self‐administered and interviewer‐assisted PROMs stratified by questionnaire (WOMAC (a), OKS (b), FJS (c)). The red line represents the line of equality. FJS, Forgotten Joint Score; OKS, Oxford Knee Score; PROMs, patient‐reported outcome measures; WOMAC, Western Ontario and McMaster Universities Osteoarthritis Index.

## DISCUSSION

The main finding of this study was that intentional interviewer influence during PROM administration consistently resulted in more favourable scores. This effect was particularly evident among patients who reported high satisfaction on the Likert scale. The observed differences can be considered against published thresholds for clinical relevance, including the minimal clinically important difference (MCID), which describes the smallest clinically relevant difference, and the minimal important change (MIC), which reflects the smallest change considered important by patients. However, these thresholds vary between questionnaires, calculation methods and follow‐up intervals [14, 24]. The FJS difference of 35.9 points clearly exceeds the reported thresholds of 10.8–14.0 points [16, 17]. The total OKS difference of 6.8 points exceeds the MCIDs of 4.3–5.0 points reported by Clement et al., based on patient‐reported improvement in function and pain relief, but remains below the 1‐year MIC of 8 points reported by Ingelsrud et al. [9, 17]. The WOMAC difference of 8.4 points remains slightly below the reported total‐score MCID of approximately 10 points [8]. Although these thresholds are not directly applicable to administration‐related differences, the comparison suggests that intentionally influenced interviewer‐assisted administration may produce differences approaching or exceeding clinically meaningful magnitudes.

Interviewer bias is a well‐recognized phenomenon in survey research and has also been reported in orthopaedic outcome assessment. Previous studies comparing different modes of administration, such as telephone, mail or in‐person completion, have yielded inconsistent results [2, 4, 13, 15, 22, 38]. A narrative review by Zini and Banfi found four papers suggesting favourable results of scores with an interviewer present, while other studies could not show this effect [38]. Rutherford et al. conducted a meta‐analysis in which they could not find relevant differences in scores by the mode of administration [30]. The present study differs from prior work by applying a within‐subject design with a defined interval and identical instruments, thereby minimizing interindividual variability and selection effects. Nevertheless, several mechanisms may contribute to the observed interviewer effect. Social desirability bias, defined as the tendency of respondents to provide answers perceived as favourable or acceptable in a social context [5], may lead patients, particularly those satisfied with their surgical outcome, to report more positive results in the presence of study personnel. In the present study, discrepancies between modes of administration were more pronounced among satisfied patients, which is consistent with the presence of social desirability effects and suggests that this mechanism may have contributed to the observed differences. Completing PROMs in a clinical environment, in close temporal association with a follow‐up visit, may positively frame the overall treatment experience and influence self‐assessment. While this has predominantly been discussed in healthcare settings with more pronounced hierarchical structures [23], it may also affect PROM collection in Western healthcare systems. Importantly, interviewer‐assisted administration in this study was not limited to neutral data collection. It included clarification of items and, in cases of uncertainty, the suggestion of the clinically more favourable response option. This active guidance likely amplified the observed interviewer effect and should be interpreted as an upper‐bound estimate of interviewer‐related influence rather than a purely passive mode effect. Consequently, the magnitude of score differences observed in this study may exceed those encountered under routine clinical conditions but clearly illustrates the susceptibility of PROMs to interviewer involvement. Beyond classical interviewer bias, response shift represents an additional explanatory mechanism. Response shift describes a change in internal standards, values or conceptualization of health that may influence how patients evaluate their condition [28, 33]. In the present study, it is conceivable that patients recalibrated their internal standards or reference frames regarding pain, function and expectations between independent completion at home and guided completion in the clinical setting, although this was not directly assessed. Such cognitive re‐evaluation may contribute to systematic differences in PROM scores independent of actual clinical change, as described in longitudinal PROM research [28, 33].

Although all evaluated PROMs were affected by interviewer‐assisted administration, the FJS showed the most pronounced susceptibility. This suggests that factors beyond general interviewer bias may be particularly relevant for this instrument. The FJS is conceptually based on the construct of ‘awareness’, representing a higher‐order cognitive state reflecting whether the joint is consciously present in everyday life. Abstract constructs such as awareness are known to be more sensitive to contextual and linguistic variation, even when appropriately translated and formally validated, as described in cross‐cultural adaptation research [3, 36]. Unlike symptom‐based measures assessing pain or functional limitation, the FJS requires a cognitive appraisal that may vary depending on individual interpretation and situational framing. This type of construct is less concrete and therefore more susceptible to subtle differences in understanding, particularly in the absence of standardized contextualization. In interviewer‐assisted settings, this may be reduced through implicit clarification, explanation or guidance when patients are uncertain between response options. Accordingly, the pronounced interviewer effect observed for the FJS may not only reflect interpersonal or social desirability bias but may also arise from differences in semantic interpretation and processing of the construct itself. This highlights that instruments assessing higher‐order cognitive concepts may be particularly vulnerable to context‐dependent influences during data collection.

### Limitations

Several limitations should be acknowledged. First, the PROMs were assessed at 10–12 weeks postoperatively, and no later follow‐up was performed. Although substantial functional recovery after TKA occurs within the first 3 months, further improvement in PROM scores may occur up to 1 year after surgery [11, 12]. Therefore, the timing of assessment may limit generalizability to later postoperative time points. Nevertheless, the PROM values observed in the present cohort were within the range of previously reported outcomes at 6 months to 1 year after TKA [25, 26, 35].

Second, as all patients completed the self‐administered PROMs before the interviewer‐assisted assessment, fixed‐order, recall and learning effects cannot be excluded, despite the approximately 2‐week interval and altered presentation of the response options. Asking patients about their satisfaction immediately before the second assessment may also have primed them toward a more favourable evaluation. Furthermore, response shift and short‐term clinical improvement between assessments may have contributed to the observed differences. However, clinical improvement alone is unlikely to explain the consistent magnitude and direction of the differences across all instruments. Third, only the German‐language versions of the PROMs were evaluated, limiting generalizability to other linguistic and cultural contexts. Finally, the interviewer‐assisted assessment deliberately included directional prompting toward more favourable responses and therefore introduced an intended source of measurement bias. Consequently, the observed differences cannot be attributed solely to the mode of administration or to item clarification, but reflect the combined effects of interviewer presence, additional explanation and intentional response prompting. As no neutral interviewer‐assisted control condition was included, the findings should not be generalized to standardized interviewer administration without directional influence. Because this intervention intentionally exceeded routine clinical practice, the observed differences likely represent an upper estimate of interviewer influence.

## CONCLUSION

PROMs after TKA are strongly influenced by the mode of administration. Interviewer‐assisted administration involving intentional influence resulted in systematically more favourable scores across all questionnaires. For meaningful comparison of PROM‐based outcomes, the mode of administration and any interviewer guidance should be standardized or at least clearly reported.

## AUTHOR CONTRIBUTIONS

*Conceptualization*: Lars‐Rene Tuecking and Mara Hold. *Data curation*: Sophie Berg, Mara Hold and Lars‐Rene Tuecking. *Formal analysis*: Mara Hold and Lars‐Rene Tuecking. *Investigation*: Sophie Berg, Lars‐Rene Tuecking, Mara Hold, Alexander Derksen, Jan Dirk Kiene, Mats Zander, Marco Haertlé and Spiros Tsamassiotis. *Methodology*: Mara Hold and Lars‐Rene Tuecking. *Project administration*: Lars‐Rene Tuecking and Henning Windhagen. *Supervision*: Lars‐Rene Tuecking and Henning Windhagen. *Visualization*: Mara Hold and Lars‐Rene Tuecking. *Writing—original draft*: Mara Hold and Lars‐Rene Tuecking. *Writing—review and editing*: Lars‐Rene Tuecking, Mara Hold, Sophie Berg, Alexander Derksen, Jan Dirk Kiene, Mats Zander, Marco Haertlé, Spiros Tsamassiotis and Henning Windhagen.

## FUNDING INFORMATION

The authors have no funding to report.

## CONFLICT OF INTEREST STATEMENT

The authors declare no conflicts of interest.

## ETHICS STATEMENT

Ethical approval for this study was obtained from the Ethics Committee of Hannover Medical School (Nr. 11678_BO_ K_2024). Written informed consent was received from every included patient.

## Supporting information

Supporting File 1.

## Data Availability

The data that support the findings of this study are available from the corresponding author upon reasonable request.
