# Supplementary material for: Interviewer‐assisted administration of patient‐reported outcome measures with intentional interviewer influence consistently produces more favourable scores than self‐administration after total knee arthroplasty: A prospective within‐subject comparative study
Source: J Exp Orthop. 2026 Oct 9;13(4):e70899. doi: 10.1002/jeo2.70899 (PMC13651008; doi:10.1002/jeo2.70899)
Supplement: Supplementary file 1 [file JEO2-13-e70899-s001.docx]

**Supplementary Table 1:** Likert-stratified paired differences between self-administered and interviewer-assisted patient-reported outcome measures

| **Questionnaire** | **Likert** | **n** | **Mean difference ± SD** | **95% CI** | **Min. diff.** | **Max. diff.** | **p-value** |
| --- | --- | --- | --- | --- | --- | --- | --- |
| **FJS** | 1 | 3 | 9.7 ± 9.6 | -14.2 to 33.6 | 4.2 | 20.8 | 0.22 |
| **FJS** | 2 | 4 | 25.1 ± 17.3 | -2.4 to 52.7 | 10.4 | 43.8 | 0.06 |
| **FJS** | 3 | 7 | 35.1 ± 8.0 | 27.7 to 42.5 | 27.1 | 47.9 | < 0.001 |
| **FJS** | 4 | 16 | 38.5 ± 15.9 | 30.0 to 47.0 | -6.3 | 66.7 | < 0.001 |
| **FJS** | 5 | 30 | 38.8 ± 18.9 | 31.7 to 45.8 | 4.2 | 75 | < 0.001 |
| **OKS** | 1 | 3 | 3.0 ± 3.6 | -5.9 to 11.9 | -1 | 6 | 0.29 |
| **OKS** | 2 | 4 | 5.3 ± 6.0 | -4.2 to 14.8 | -3 | 11 | 0.18 |
| **OKS** | 3 | 7 | 8.6 ± 2.4 | 6.4 to 10.8 | 6 | 13 | < 0.001 |
| **OKS** | 4 | 16 | 9.9 ± 5.3 | 7.1 to 12.7 | 2 | 20 | < 0.001 |
| **OKS** | 5 | 30 | 5.3 ± 3.9 | 3.8 to 6.8 | -5 | 16 | < 0.001 |
| **WOMAC** | 1 | 3 | -0.7 ± 13.4 | -34.0 to 32.6 | -15.4 | 10.9 | 0.94 |
| **WOMAC** | 2 | 4 | -9.5 ± 10.6 | -26.3 to 7.4 | -22.9 | 2.1 | 0.17 |
| **WOMAC** | 3 | 7 | -15.9 ± 11.4 | -26.4 to -5.4 | -35.4 | 0 | 0.010 |
| **WOMAC** | 4 | 16 | -10.4 ± 14.6 | -18.2 to -2.6 | -45.0 | 20.8 | 0.012 |
| **WOMAC** | 5 | 30 | -6.1 ± 7.2 | -8.8 to -3.4 | -29.6 | 3.8 | < 0.001 |

Differences were calculated as interviewer-assisted minus self-administered scores. Positive FJS and OKS differences and negative WOMAC differences indicate more favorable scores during interviewer-assisted administration. CI, confidence interval; diff., difference; FJS, Forgotten Joint Score; max., maximum; min., minimum; n, number of patients; OKS, Oxford Knee Score; p, probability value; PROMs, patient-reported outcome measures; SD, standard deviation; WOMAC, Western Ontario and McMaster Universities Osteoarthritis Index. Data are presented as mean difference ± SD. The 95% CI represents the 95% confidence interval of the mean difference.

**Supplementary Figure 1:** Scatterplots comparing self-administered and interviewer-assisted questionnaires results stratified by postoperative satisfaction (Likert 1-5). The red line indicates equality.

FJS, Forgotten Joint Score; OKS, Oxford Knee Score; PROMs, patient-reported outcome measures; WOMAC, Western Ontario and McMaster Universities Osteoarthritis Index.
